# Supplementary material for: Miro1-dependent mitochondrial dynamics in parvalbumin interneurons
Source: eLife. 2021 Jun 30;10:e65215. doi: 10.7554/eLife.65215 (PMC8294849; doi:10.7554/eLife.65215)
Supplement: Transparent reporting form [file elife-65215-transrepform.docx]

***eLife’s* transparent reporting form**

We encourage authors to provide detailed information *within their submission* to facilitate the interpretation and replication of experiments. Authors can upload supporting documentation to indicate the use of appropriate reporting guidelines for health-related research (see [EQUATOR Network](http://www.equator-network.org/%20)), life science research (see the [BioSharing Information Resource](https://biosharing.org/)), or the [ARRIVE guidelines](http://www.plosbiology.org/article/info:doi/10.1371/journal.pbio.1000412) for reporting work involving animal research. Where applicable, authors should refer to any relevant reporting standards documents in this form.

If you have any questions, please consult our Journal Policies and/or contact us: [editorial@elifesciences.org](mailto:editorial@elifesciences.org).

**Sample-size estimation**

- You should state whether an appropriate sample size was computed when the study was being designed
- You should state the statistical method of sample size computation and any required assumptions
- If no explicit power analysis was used, you should describe how you decided what sample (replicate) size (number) to use

Please outline where this information can be found within the submission (e.g., sections or figure legends), or explain why this information doesn’t apply to your submission:

The power analysis was not used to estimate sample sizes. Estimates of sample sizes were based on previous work (Stephen et al. 2015; López-Doménech et al. 2016; Antonoudiou et al. 2020). Detailed n numbers for each individual experiment can be found in the figure legends. Briefly, for the mitochondrial trafficking experiments, n_Miro(+/+)_ = 15 movies, 9 slices from 4 animals, n_Miro(+/Δ)_ = 32 movies, 10 slices from 4 animals and n_Miro(Δ/Δ)_ = 22 movies, 8 slices from 4 animals. For the morphological reconstructions, 5 neurons per group were used. The cells which were accessible for filling were usually closer to the surface of the brain slice and the dendrites or the axon were cut most of the times, so the probability of finding intact cells was very low. For channel rhodopsin photostimulation experiments, n_Miro(+/+)_ = 21-23 recordings from 4 animals and n_Miro(Δ/Δ)_ = 18-24 recordings from 4 animals. For LFP experiments (for γ-oscillations) n_Miro(+/+)_ = 12 recordings from 2 animals, n_Miro(+/Δ)_ = 23 recordings from 6 animals and n_Miro(Δ/Δ)_ = 20 recordings from 6 animals. For animal behaviour ~8 animals were used per group (except for motor coordination experiments where 5 animals per group were used).

**Replicates**

- You should report how often each experiment was performed
- You should include a definition of biological versus technical replication
- The data obtained should be provided and sufficient information should be provided to indicate the number of independent biological and/or technical replicates
- If you encountered any outliers, you should describe how these were handled
- Criteria for exclusion/inclusion of data should be clearly stated
- High-throughput sequence data should be uploaded before submission, with a private link for reviewers provided (these are available from both GEO and ArrayExpress)

Please outline where this information can be found within the submission (e.g., sections or figure legends), or explain why this information doesn’t apply to your submission:

The reported n values are for biological replicates, which represent either the number of mitochondria, number of neurons, number of slices, number of movies/recordings and number of animals based on the experiment. This information can be found in the figure legends. Statistical outliers were identified using the ROUT method and removed as described by Motulsky, H.J., and Brown, R.E. (2006). **Detecting outliers when fitting data with nonlinear regression - a new method based on robust nonlinear regression and the false discovery rate**. *BMC Bioinformatics 7, 123.* This information can be found in the Methods under the Statistical Analysis and Blinding section.

**Statistical reporting**

- Statistical analysis methods should be described and justified
- Raw data should be presented in figures whenever informative to do so (typically when N per group is less than 10)
- For each experiment, you should identify the statistical tests used, exact values of N, definitions of center, methods of multiple test correction, and dispersion and precision measures (e.g., mean, median, SD, SEM, confidence intervals; and, for the major substantive results, a measure of effect size (e.g., Pearson's r, Cohen's d)
- Report exact p-values wherever possible alongside the summary statistics and 95% confidence intervals. These should be reported for all key questions and not only when the p-value is less than 0.05.

Please outline where this information can be found within the submission (e.g., sections or figure legends), or explain why this information doesn’t apply to your submission:

The statistical tests used are identified in the main text. The exact *p* values are reported in the text and represented as *p < 0.05, **p < 0.01, ****p < 0.001 and ****p < 0.0001. The detailed n numbers are found in the figure legends. The bar charts show the mean ± standard error of the mean (sem). The boxplots show the min to max box-and-whisker with the middle line plotted at the median.

(For large datasets, or papers with a very large number of statistical tests, you may upload a single table file with tests, Ns, etc., with reference to sections in the manuscript.)

**Group allocation**

- Indicate how samples were allocated into experimental groups (in the case of clinical studies, please specify allocation to treatment method); if randomization was used, please also state if restricted randomization was applied
- Indicate if masking was used during group allocation, data collection and/or data analysis

Please outline where this information can be found within the submission (e.g., sections or figure legends), or explain why this information doesn’t apply to your submission:

Animals were allocated into experimental groups based on their genotype (Miro^+/+^, Miro^+/Δ^ and Miro^Δ/Δ^. Masking/blinding was used during brain tissue processing, staining, acquisition and analysis. Live-imaging acquisition and analysis were also performed blinded for littermate Miro^+/Δ^ and Miro^Δ/Δ^ animals. Electrophysiological recording analysis was performed blinded. This information can be found in the Methods under the Statistical Analysis and Blinding section.

**Additional data files (“source data”)**

- We encourage you to upload relevant additional data files, such as numerical data that are represented as a graph in a figure, or as a summary table
- Where provided, these should be in the most useful format, and they can be uploaded as “Source data” files linked to a main figure or table
- Include model definition files including the full list of parameters used
- Include code used for data analysis (e.g., R, MatLab)
- Avoid stating that data files are “available upon request”

Please indicate the figures or tables for which source data files have been provided:

An excel file with the extended statistical tests for significant data (Fig1A, Fig1C, Fig1D, Fig1E, Fig2B, Fig2C, Fig2G, Fig2J, Fig3C, Fig3D, Fig3F, Fig3K, Fig5C, Fig5F, Fig5H, Fig5I, Fig5K, S.Fig1D, S.Fig2C and S.Fig2G).
